# Correlation between the Systemic Immunoinflammatory Index and Platelet–Lymphocyte Ratio in Patients with Adenomyosis

**DOI:** 10.1155/2024/9977750

**Published:** 2024-01-17

**Authors:** Yan Dong, YaHui Chen, YaNan Wang, Lin Wang, Yan Zhou, Mei Xue, Lin Sun

**Affiliations:** ^1^Department of Gynecology and Obstetrics, Affiliated Hospital of Jining Medical University, Jining 272029, Shandong, China; ^2^Jining Medical University, Jining 272002, Shandong, China

## Abstract

**Background:**

The chronic inflammatory immune response is a significant factor in the pathogenesis of benign gynecological diseases. The systemic immunoinflammatory index (SII) and the platelet-to-lymphocyte ratio (PLR) are commonly available biomarkers of inflammation. However, evidence of the relationship between SII and PLR in patients with adenomyosis is limited. This study aimed to investigate the relationship between SII and PLR in patients with adenomyosis.

**Methods:**

This cross-sectional study included 483 patients with adenomyosis who were first diagnosed at our institution between January 2019 and December 2021. Basic patient clinical information and inflammatory factors were collected for univariate analysis, smoothed curve fitting, and multivariate segmented linear regression.

**Results:**

The results of the univariate analysis showed a significant positive correlation between PLR levels and SII (*P* < 0.001). In addition, a nonlinear relationship between PLR and SII was tested using a smoothed curve fit after adjusting for potential confounders. Multiple segmented linear regression models showed a significant relationship between SII and PLR in both SII < 1,326.47 (*β* 0.14, 95% CI: 0.12, 0.16; *P* < 0.0001) and >1,326.47 (*β* 0.02, 95% CI: −0.01, 0.05; *P* = 0.2461).

**Conclusions:**

In conclusion, this study showed a nonlinear relationship between SII and PLR in patients with uterine adenomyosis. An increase in serum PLR levels correlates with an increase in SII before SII levels reach an inflection point.

## 1. Introduction

Adenomyosis is a condition in which the endometrium (including glands and mesenchyme) invades the myometrium and grows into the uterus [1]. The occurrence of adenomyosis is related to childbirth, including abortion, curettage, and other trans-uterine operations or surgeries that may cause damage to the endometrium, making the endometrium depressed or invading the myometrium, thus leading to the occurrence of adenomyosis [2, 3]. Related studies have identified a large number of cytokines, chemokines, growth factors, and prostaglandins as key promoters in the initiation, maintenance, and progression of benign gynecological disease, and adenomyosis may also induce local and systemic inflammation [4].

The systemic immunoinflammatory index (SII) is defined as neutrophil count × platelet count/lymphocyte count [5]. SII is a new indicator of systemic immunoinflammation that has been proposed in recent years and is a prognostic indicator for a variety of cancers [6]. High-SII levels may be associated with shorter overall survival in malignancies such as pancreatic, gynecological, and breast cancers [7]. Platelet-to-lymphocyte ratio (PLR) is defined as platelet count/lymphocyte count. PLR may be associated with poor prognosis in some solid tumors such as oesophageal, colorectal, hepatocellular, cervical, breast, and pancreatic cancers [8, 9]. SII and PLR represent the balance between neutrophils, lymphocytes, and platelets [10, 11]. Recently, SII and PLR have received a lot of attention as new indicators of local and systemic immune status [9]. SII levels are of very high-diagnostic value in differentiating the severity of the disease course caused by the SARS-CoV-2 virus [12]. SII levels are highly correlated with the depth of trophoblastic infiltration in tubal ectopic pregnancy and can also be used to predict tubal ectopic pregnancy rupture [13]. Significantly, higher levels of SII and PLR in patients with preeclampsia compared to healthy pregnant women can be used to predict inflammatory status in patients with preeclampsia [14]. However, the relationship between SII and PLR and patients with adenomyosis is unclear. This study aimed to elucidate the relationship between SII and PLR in patients with adenomyosis.

## 2. Materials and Methods

### 2.1. Patients and Clinicopathological Data

This single-center cross-sectional study was approved by the Institutional Review Board of the Jining Medical University Hospital (approval number: 2022C114). Due to the retrospective nature of the cohort study, informed consent was not required.

**Figure 1:**
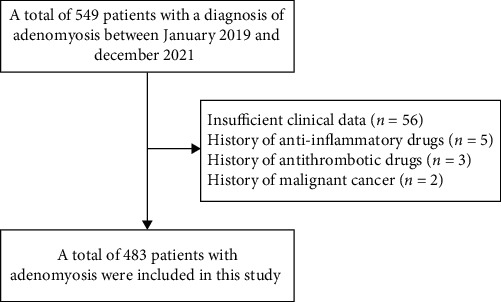
Flowchart of patient selection.

The study population was patients diagnosed with adenomyosis from January 2019 to December 2021 at the Department of Gynecology, Affiliated Hospital of Jining Medical University (Figure 1). Inclusion criteria were as follows: (1) pathologically diagnosed with adenomyosis; (2) well-documented clinical cases. Exclusion criteria were as follows: (1) patients with hematological disorders, malignancies, autoimmune diseases, metabolic diseases, or existing infections; (2) patients treated with hormone, glucocorticoids, permanent immunomodulatory drugs, antithrombotic drugs, or anti-inflammatory drugs in the 3 months before admission; (3) patients under 18 years of age; (4) women in pregnancy and menstruation; (5) history of previous treatment for adenomyosis.

These data did not include identifiable information to protect patient privacy and were obtained from the hospital's electronic medical record system. A total of 483 patients were enrolled.

### 2.2. Evaluation of Clinical Characteristics and Determination of Blood Parameters

Data were collected retrospectively from patients, including age, body mass index (BMI), menstrual status, gravidity, parity, uterine size, and routine blood indicators. Anaemia was defined as hemoglobin below 110, 90–110 g/L as mild anemia, 60–90 g/L as moderate anemia, and less than 60 g/L as severe anemia. The history is taken to obtain the patient's gravidity, parity, menstrual regularity, menstrual volume, and whether the patient is menopausal. Dysmenorrhea is pain and swelling in the lower abdomen around the time of menstruation or during menstruation, accompanied by back pain. Menopause is the permanent termination of physiological menstruation. The criteria for regularity of menstruation was a menstrual regularity of 21–35 days and a period of 4–6 days. Those who met the criteria were considered regular and those who did not were considered irregular. Menstrual volume was judged to be less than 20, 20–80 mL normal, and more than 80 mL excessive. The volume of the uterus is calculated as long diameter× wide diameter × anterior–posterior diameter × *π*/6. Fasting blood samples were obtained from all subjects for measurement of laboratory parameters: peripheral venous blood was collected after 8 hr of fasting on admission and routine blood was measured using a Sysmex XN2000 hematocrit analyzer, from which we obtained platelet count, lymphocyte count, and neutrophil count for the calculation of SII and PLR. All measurements were performed by the hospital laboratory technicians and examiners. SII was neutrophil count × platelet count/lymphocyte count and PLR was the ratio of platelet to lymphocyte count, using the same blood samples.

### 2.3. Statistical Analysis

All statistical analyses were performed with R statistical software (https://www.r-project.org) and Empower Stats (http://www.empowerstats.com, X&Y Solutions, Inc. Boston MA). Normally distributed continuous variables are expressed as mean ± standard deviation and nonnormally distributed continuous variables are expressed as median. Categorical variables are expressed as frequencies or percentages. For further analyses, the SII was equally divided into four groups, ranging from the lowest quartile to the highest quartile, A *χ*^2^ test (for categorical variables), a one-way ANOVA test (for normal distribution), or a Kruskal–Wallis test (for skewed distribution) were used to test for differences between the different SII groups. The whole data analysis process can be divided into two steps. Step 1: a multivariate linear regression model adjusted for patient characteristics and significant variables from the univariate analysis was created. Step 2: a generalized additive model and a smoothed curve fit (penalized spline method) were developed for the nonlinearity of SII and PLR. If nonlinearity is detected, we first calculate the inflection point using a recursive algorithm and then construct a two-segment linear regression on either side of the inflection point. We determined the best-fit model based on the *P*-value of the log-likelihood ratio test. To ensure the robustness of the data analysis, we performed a sensitivity analysis. We transformed SII into a categorical variable and calculated the *P* for trend. The aim was to validate the results for SII as a continuous variable and to observe the possibility of nonlinearity. Statistical significance was accepted at a two-sided *P*-value < 0.05.

## 3. Results

### 3.1. Baseline Characteristics of Included Patients

A total of 483 participants were included in the final analysis. Baseline patient characteristics according to SII quartiles are shown in Table 1. The mean age of the patients was 46.39 ± 5.64 *y*. There were no statistically significant differences between the SII groups for age, BMI, gravidity, menstrual regularity, dysmenorrhea, hypertension, benign adnexal tumor, endometriosis, uterine leiomyoma, and uterine adenomyoma (*P* > 0.05). Also, there was a significant difference in menstrual volume, uterine volume, uterine length/width/thickness, diabetes, menopause, and anemia (*P* < 0.05). Participants in the highest SII quartile (Q4) had higher platelet counts, neutrophil counts, and lower lymphocyte count, and hemoglobin.

### 3.2. Factors Associated with PLR of the Study Population

The results of the univariate analysis are shown in Table 2. Univariate analysis showed that age, BMI, gravidity, parity, menstrual regularity, dysmenorrhoea, menstrual volume, endometriosis, uterine leiomyoma, adenomyoma, benign adnexal tumors, uterine volume, and uterine length/width/thickness were not associated with PLR. In addition, univariate analysis showed that hypertension, diabetes, menopause, anemia, Hemoglobin, and SII were positively associated with PLR (*P* < 0.05).

### 3.3. Results of Adjusted Linear Regression

Models for confounding factors were developed using multiple linear regression to analyze the independent effects of SII on PLR, the results are shown in Table 3. SII and PLR were significantly correlated in unadjusted (Model I) and adjusted (Model II and Model III) models. For example, in the unadjusted model (Model I), when SII increased by 1 unit, PLR increased by 0.11 unit (*β* 0.11, 95% CI: 0.10, 0.12). For further sensitivity analysis, SII was converted from a continuous variable to a categorical variable (SII quartiles). The *P*-values for the PLR trend in the minimally adjusted and fully adjusted models were consistent with the *P*-values when SII was a continuous variable.

Adjust II adjusts for age, BMI, gravidity, parity, menstrual regularity, dysmenorrhoea, menstrual volume, uterine leiomyoma, adenomyoma, hypertension, diabetes, endometriosis, benign adnexal tumors, uterine volume, uterine length/width/thickness, hemoglobin and menopause.

Adjust III adjusts for age, BMI, gravidity, parity, menstrual regularity, dysmenorrhoea, menstrual volume, uterine leiomyoma, adenomyoma, hypertension, diabetes, endometriosis, benign adnexal tumors, uterine volume, uterine length/width/thickness, menopause, anemia, and hemoglobin.

### 3.4. The Results of Nonlinearity of the SII and PLR

**Figure 2:**
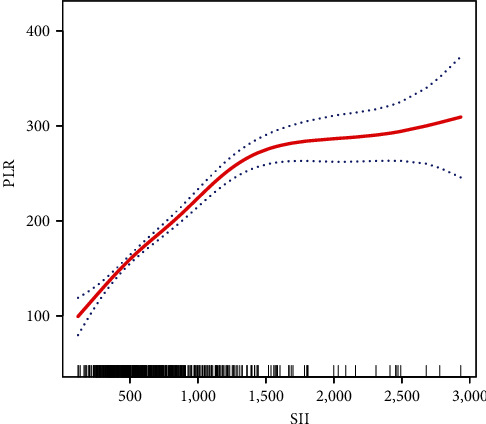
Relationship between SII and PLR by smooth curve fitting. The smooth fitting curve of SII and PLR. Adjustment variables: age, BMI, gravidity, parity, menstrual regularity, dysmenorrhoea, menstrual volume, uterine leiomyoma, adenomyoma, hypertension, diabetes, endometriosis, benign adnexal tumors, uterine volume, uterine length/width/thickness, menopause, anemia, and hemoglobin.

As shown in Figure 2, smoothed curve fits were performed after adjusting for the possible confounders including age, BMI, gravidity, parity, menstrual regularity, dysmenorrhoea, menstrual volume, uterine leiomyoma, adenomyoma, hypertension, diabetes, endometriosis, benign adnexal tumors, uterine volume, uterine length/width/thickness, menopause, anemia, and hemoglobin. PLR has a nonlinear relationship with SII. Specifically, PLR levels increased with increasing SII. As shown in Table 4, the threshold effect was further analyzed based on curve fitting, which showed that the inflection point for SII was 1,326.47. When SII was less than 1,326.47, PLR levels increased with increasing SII (*β* 0.14, 95% CI: 0.12, 0.16; *P* < 0.0001). When SII was greater than 1,326.47, PLR levels increased with increasing SII (*β* 0.02, 95% CI: −0.01, 0.05; *P*=0.2461), however, there was no statistical significance. Differential linear regression and segmented linear regression were assessed by a log-likelihood ratio test. *P*-values less than 0.05 implied that the segmented linear function was better for observation; otherwise, the linear function was better. The results indicate that the *P* for the log-likelihood ratio test is less than 0.05; therefore, the double-segmented linear regression used to fit the association between SII and PLR accurately represents this relationship.

**Figure 3:**
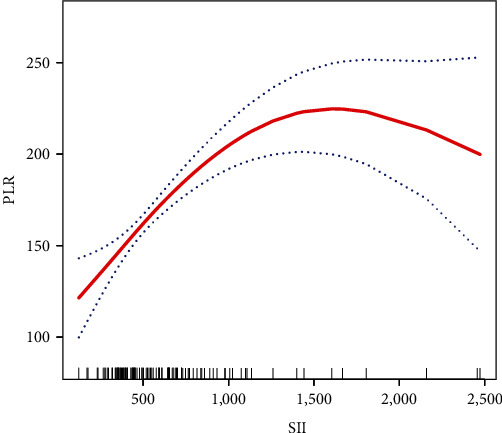
Relationship between SII and PLR in patients with combined adenomyoma by smooth curve fitting. Adjustment variables: age, BMI, gravidity, parity, menstrual regularity, dysmenorrhoea, menstrual volume, uterine leiomyoma, hypertension, diabetes, endometriosis, benign adnexal tumors, uterine volume, uterine length/width/thickness, menopause, anemia, and hemoglobin.

There was an independent correlation between SII and PLR in patients with combined adenomyoma. The smoothed fitted curves for patients with combined adenomyoma are shown in Figure 3.

## 4. Discussion

This cross-sectional study observed a nonlinear relationship between SII and PLR in patients with adenomyosis, with an SII inflection point of 1,326.47 mg/dL. When SII was less than 1,326.47 mg/dL, there was a significant positive correlation between SII and PLR.

SII, a novel inflammatory response biomarker based on neutrophil, lymphocyte, and platelet counts, integrates the kinetics of NLR and PLR into a single parameter that provides a comprehensive response to the balance between host immunity and inflammatory status [6, 15, 16]. An increasing amount of research has shown that SII is involved in the prognosis of various malignancies, including colorectal, oesophageal, and pancreatic cancers [17, 18]. High levels of SII are significantly associated with the risk of recurrence and with risk of death in a population of patients with early stage ovarian cancer [19]. PLR is a newly recognized marker of inflammation that can be used as a prognostic indicator for a variety of diseases, including COVID-19, endometrial cancer, ovarian cancer, and endometriosis [20–22]. Neutrophils provide biologically active molecules needed in tumor progression through proangiogenesis, and animal studies have shown that neutrophils in mice with endometriosis can induce new lesion formation [23].

A study found that peripheral blood PLR levels were higher in patients with endometrial cancer and in patients with benign endometrial hyperplasia than in the normal group of patients [24, 25]. Activated platelets promote invasion by cell migration and increased collagen production, leading to increased fibrosis in adenomyosis [26, 27]. Animal studies have shown a positive correlation between the degree of ectopic endometrial platelet aggregation and the medium microvascular density of adenomyosis lesions [26]. A study found that platelet count (PLT) was higher in patients with adenomyosis than in patients with uterine smooth muscle tumors [28]. PLT was positively correlated with pelvic adhesions in patients with adenomyosis and combined with CA125 played an important role in the diagnosis and assessment of adhesions in adenomyosis [29, 30]. One study reported that changes in lymphocytes and their associated factors in patients with adenomyosis were associated with the release of proinflammatory mediators, pelvic pain in adenomyosis, and infertility [31, 32].

Recent studies have shown that immune dysregulation and inflammation occur in patients with adenomyosis and that high expression of inflammatory factors and neurogenic mediators in adenomyotic lesions contributes to the persistence and growth of endometrial implants involved in the development and progression of adenomyosis [33–35]. In this study, we analyzed the relationship between SII and PLR in patients with adenomyosis by smoothing curve fitting and observed a nonlinear relationship between SII and PLR, with PLR levels increasing with increasing SII. This finding suggests that SII and PLR can respond to the inflammatory state of the body and that there is also a positive correlation between SII and PLR in patients with combined adenomyosis.

On the other hand, there are several limitations to the current study. First, the nature of the cross-sectional analysis in this study, without a control group, does not allow us to infer a causal relationship, and further investigation is needed to follow changes in SII and PLR to determine whether this relationship is maintained or changed after adenomyosis treatment. Second, our study was conducted on patients diagnosed with adenomyosis and the results cannot be generalized to other populations. Third, as this study was a single-center study and the participants were mainly Chinese Han adult women, future multicenter studies are planned to analyze the relationship between PLR and SII in patients with adenomyosis, and more studies are needed to explain the potential mechanisms between SII and PLR.

## 5. Conclusion

In conclusion, the present study describes a nonlinear relationship between SII and PLR in patients with adenomyosis after adjusting for the potential confounding factors. An increase in serum PLR levels correlates with an increase in SII before SII levels reach an inflection point. The results will contribute to understanding the relationship between SII and PLR in patients with adenomyosis. However, a causal relationship between SII and PLR could not be established and further studies are needed to determine the possible mechanism.

## Figures and Tables

**Table 1:** Baseline characteristics of included patient by SII quartile.

Characteristic	SII (min–max)				*P* value
	Q1 (118.33–403.04)	Q2 (403.50–571.32)	Q3 (575.96–841.21)	Q4 (842.00–2933.89)	
*N*	116	123	124	120	
Age (years, mean ± SD)	46.53 ± 5.65	46.41 ± 5.65	46.54 ± 5.75	46.09 ± 5.59	0.921
Gravidity (*n*)	3 (0–9)	3 (1–8)	3 (0–8)	3 (0–9)	0.059
Parity (*n*)	2 (0–4)	2 (1–5)	2 (0–4)	2 (0–4)	0.176
Gravidity (*n*, %)					0.515
0	1 (0.86%)	0 (0.00%)	4 (3.23%)	2 (1.67%)	
1	8 (6.90%)	8 (6.50%)	10 (8.06%)	8 (6.67%)	
2 and more	107 (92.24%)	115 (93.50%)	110 (88.71%)	110 (91.67%)	
Parity (*n*, %)					0.016
0	1 (0.86%)	0 (0.00%)	7 (5.65%)	3 (2.50%)	
1	54 (46.55%)	47 (38.21%)	48 (38.71%)	38 (31.67%)	
2 and more	61 (52.59%)	76 (61.79%)	69 (55.65%)	79 (65.83%)	
BMI (kg/m^2^, mean ± SD)	24.70 ± 3.11	27.79 ± 26.88	25.16 ± 3.15	24.74 ± 3.34	0.248
Menstrual regularity, *n* (%)					0.362
Regular	111 (95.69%)	114 (92.68%)	120 (96.77%)	111 (92.50%)	
Irregular	5 (4.31%)	9 (7.32%)	4 (3.23%)	9 (7.50%)	
Dysmenorrhea, *n* (%)					0.416
No	57 (49.14%)	61 (49.59%)	50 (40.32%)	58 (48.33%)	
Yes	59 (50.86%)	62 (50.41%)	74 (59.68%)	62 (51.67%)	
Menstrual volume					0.019
Low	5 (4.31%)	2 (1.63%)	4 (3.23%)	1 (0.83%)	
Moderate	80 (68.97%)	80 (65.04%)	74 (59.68%)	61 (50.83%)	
High	31 (26.72%)	41 (33.33%)	46 (37.10%)	58 (48.33%)	
Hypertension, *n* (%)					0.217
No	100 (86.21%)	112 (91.06%)	106 (85.48%)	111 (92.50%)	
Yes	16 (13.79%)	11 (8.94%)	18 (14.52%)	9 (7.50%)	
Diabetes, *n* (%)					0.025
No	116 (100.00%)	123 (100.00%)	119 (95.97%)	118 (98.33%)	
Yes	0 (0.00%)	0 (0.00%)	5 (4.03%)	2 (1.67%)	
Endometriosis, *n* (%)					0.416
No	100 (86.21%)	103 (83.74%)	97 (78.23%)	98 (81.67%)	
Yes	16 (13.79%)	20 (16.26%)	27 (21.77%)	22 (18.33%)	
Benign adnexal tumor, *n* (%)					0.076
No	68 (58.62%)	63 (51.22%)	80 (64.52%)	60 (50.00%)	
Yes	48 (41.38%)	60 (48.78%)	44 (35.48%)	60 (50.00%)	
Uterine leiomyoma					0.897
No	49 (42.24%)	53 (43.09%)	49 (39.52%)	53 (44.17%)	
Yes	67 (57.76%)	70 (56.91%)	75 (60.48%)	67 (55.83%)	
Uterine adenomyoma					0.356
No	85 (73.28%)	100 (81.30%)	96 (77.42%)	98 (81.67%)	
Yes	31 (26.72%)	23 (18.70%)	28 (22.58%)	22 (18.33%)	
Menopause					<0.001
No	104 (89.66%)	117 (95.12%)	123 (99.19%)	120 (100.00%)	
Yes	12 (10.34%)	6 (4.88%)	1 (0.81%)	0 (0.00%)	
Anaemia					<0.001
Normal	67 (57.76%)	46 (37.40%)	42 (33.87%)	23 (19.17%)	
Mild	27 (23.28%)	41 (33.33%)	36 (29.03%)	39 (32.50%)	
Moderate	21 (18.10%)	34 (27.64%)	40 (32.26%)	52 (43.33%)	
Severe	1 (0.86%)	2 (1.63%)	6 (4.84%)	6 (5.00%)	
Uterine volume (cm^3^)	184.79 ± 129.50	193.84 ± 108.61	220.21 ± 170.14	241.00 ± 150.91	0.009
Length (cm)	7.09 ± 1.81	7.20 ± 1.45	7.38 ± 1.78	7.72 ± 1.81	0.028
Width (cm)	6.92 ± 1.77	7.07 ± 1.44	7.35 ± 1.84	7.61 ± 1.68	0.010
Thickness (cm)	6.21 ± 1.65	6.58 ± 1.51	6.66 ± 1.71	6.95 ± 1.62	0.006
Hemoglobin	112.66 ± 21.93	102.41 ± 21.74	100.09 ± 25.61	90.39 ± 20.49	<0.001
Platelet (10^9^/L, mean ± SD)	241.68 ± 53.80	281.20 ± 62.26	307.72 ± 76.08	354.57 ± 94.31	<0.001
Neutrophil (10^9^/L, mean ± SD)	2.75 ± 0.89	3.23 ± 0.97	4.07 ± 1.06	5.46 ± 2.16	<0.001
Lymphocyte (10^9^/L, mean ± SD)	2.10 ± 0.57	1.85 ± 0.54	1.79 ± 0.59	1.47 ± 0.41	<0.001
SII (mean ± SD)	312.77 ± 68.19	480.99 ± 46.65	694.64 ± 77.47	1306.82 ± 468.09	<0.001
PLR (mean ± SD)	120.84 ± 34.71	161.07 ± 48.00	181.17 ± 46.56	255.46 ± 85.26	<0.001

**Table 2:** Univariate analysis of PLR.

Covariate	Statistics	*β* (95% CI)	*P* value
Age (years)	46.39 ± 5.64	0.18 (−1.00, 1.37)	0.7608
BMI (kg/m^2^, mean ± SD)	25.61 ± 13.86	−0.21 (−0.69, 0.27)	0.3988
Gravidity (*n*)	3(0–9)	−2.18 (−6.64, 2.28)	0.3386
Parity (*n*)	2 (0–5)	3.29 (−6.01, 12.59)	0.4888
Gravidity (*n*, %)			
0	7 (1.45%)	Reference	
1	34 (7.04%)	−32.96 (−93.72, 27.81)	0.2883
2 and more	442 (91.51%)	−30.42 (−86.19, 25.36)	0.2856
Parity (*n*, %)			
0	11 (2.28%)	Reference	
1	187 (38.72%)	−18.25 (−63.60, 27.10)	0.4306
2 and more	285 (59.01%)	−7.13 (−52.04, 37.78)	0.7559
Menstrual regularity, *n* (%)			
Regular	456 (94.41%)	Reference	
Irregular	27 (5.59%)	17.37 (−11.59, 46.33)	0.2404
Dysmenorrhea, *n* (%)			
No	226 (46.79%)	Reference	
Yes	257 (53.21%)	8.16 (−5.17, 21.50)	0.2307
Menstrual volume			
Low	12 (2.48%)	Reference	
Moderate	295 (61.08%)	18.79 (−23.70, 61.28)	0.3865
High	176 (36.44%)	44.82 (1.78, 87.86)	0.0418
Hypertension, *n* (%)			
No	429 (88.82%)	Reference	
Yes	54 (11.18%)	−26.49 (−47.50, −5.48)	0.0138
Diabetes, *n* (%)			
No	476 (98.55%)	Reference	
Yes	7 (1.45%)	75.50 (20.15, 130.84)	0.0078
Endometriosis, *n* (%)			
No	398 (82.40%)	Reference	
Yes	85 (17.60%)	−4.21 (−21.70, 13.28)	0.6374
Benign adnexal tumor, *n* (%)			
No	271 (56.11%)	Reference	
Yes	212 (43.89%)	13.34 (−0.03, 26.72)	0.0511
Uterine Leiomyoma			
No	204 (42.24%)	Reference	
Yes	279 (57.76%)	−0.08 (−13.57, 13.41)	0.9903
Uterine adenomyoma			
No	379 (78.47%)	Reference	
Yes	104 (21.53%)	−12.30 (−28.48, 3.87)	0.1366
Uterine volume (cm^3^)	210.15 ± 143.12	0.04 (−0.01, 0.09)	0.0847
Length (cm)	7.35 ± 1.73	3.83 (−0.01, 7.66)	0.0513
Width (cm)	7.24 ± 1.71	3.80 (−0.09, 7.70)	0.0563
Thickness (cm)	6.61 ± 1.64	3.97 (−0.09, 8.03)	0.0561
Menopause			
No	464 (96.07%)	Reference	
Yes	19 (3.93%)	−57.65 (−91.54, −23.76)	0.0009
Anaemia			
Normal	178 (36.85%)	Reference	
Mild	143 (29.61%)	42.68 (27.78, 57.58)	<0.0001
Moderate	147 (30.43%)	65.80 (51.01, 80.58)	<0.0001
Severe	15 (3.11%)	126.78 (91.12, 162.44)	<0.0001
Hemoglobin	101.29 ± 23.81	−1.34 (−1.60, −1.09)	<0.0001
SII (mean ± SD)	700.61 ± 444.18	0.11 (0.10, 0.12)	<0.0001

**Table 3:** Relationship between SII and PLR in different models.

Variable	Unadjusted Model I		Adjusted Model II		Adjusted Model III	
	*β* (95% CI)	*P*-value	*β* (95% CI)	*P*-value	*β* (95% CI)	*P*-value
SII	0.11 (0.10, 0.12)	<0.0001	0.10 (0.09, 0.11)	<0.0001	0.10 (0.09, 0.11)	<0.0001
SII (quartile)						
Q1 (118.33–403.04)	Reference		Reference		Reference	
Q2 (403.50–571.32)	40.23 (25.80, 54.67)	<0.0001	32.39 (18.21, 46.57)	<0.0001	33.40 (19.34, 47.46)	<0.0001
Q3 (575.96–841.21)	60.33 (45.93, 74.74)	<0.0001	49.57 (35.04, 64.09)	<0.0001	49.49 (35.12, 63.86)	<0.0001
Q4 (842.00–2933.89)	134.62 (120.10, 149.15)	<0.0001	117.87 (102.81, 132.93)	<0.0001	119.43 (104.48, 134.37)	<0.0001

**Table 4:** Threshold effect analysis for the relationship between the SII and PLR.

	PLR	
	Adjusted *β* value (95% CI)	*P*-value
Model I		
One linear effect	0.10 (0.09, 0.11)	<0.0001
Model II		
Breakpoint (*k*)	1,326.47	
<1,326.47 segment effect 1	0.14 (0.12, 0.16)	<0.0001
>1,326.47 segment effect 2	0.02 (−0.01, 0.05)	0.2461
LRT test	<0.001	

*Note*. Model I, linear analysis; Model II, nonlinear analysis. LRT test, logarithmic likelihood ratio test. (*P*-value < 0.05 means Model II is significantly different from Model I, which indicates a nonlinear relationship). Adjustment variables: age, BMI, gravidity, parity, menstrual regularity, dysmenorrhoea, menstrual volume, uterine leiomyoma, adenomyoma, hypertension, diabetes, endometriosis, benign adnexal tumors, uterine volume, uterine length/width/thickness, menopause, anemia, and hemoglobin.

## Data Availability

Data will be made available on request.
