# Correction to “Correlation between the Systemic Immunoinflammatory Index and Platelet–Lymphocyte Ratio in Patients with Adenomyosis”

**DOI:** 10.1155/mi/9870832

**Published:** 2025-10-27

Y. Dong, Y. Chen, Y. Wang, et al., “Correlation between the Systemic Immunoinflammatory Index and Platelet–Lymphocyte Ratio in Patients with Adenomyosis,” *Mediators of Inflammation* 2024 (2024): 9977750, https://doi.org/10.1155/2024/9977750.

In the article, there is an error in the ethical approval code stated in the Materials and Methods section. The correct ethical approval code is 2022C231.

Additionally, there is an error in the abstract:

“Multiple segmented linear regression models showed a significant relationship between SII and PLR in both SII < 1326.47 (*β* 0.14, 95% CI: 0.12, 0.16; *p* < 0.0001) and >1326.47 (*β* 0.02, 95% CI: −0.01, 0.05; *p*=0.2461).”

It should be read as follows:

“Multiple segmented linear regression models showed a significant relationship between SII and PLR in both SII < 1326.47 (*β* 0.14, 95% CI: 0.12, 0.16; *p* < 0.0001), however, we did not observe a significant relationship between SII and PLR when SII >1326.47 (*β* 0.02, 95% CI: −0.01, 0.05; *p*=0.2461).”

We apologize for this error.

